# Perceived efficacy of e-cigarettes versus nicotine replacement therapy among successful e-cigarette users: a qualitative approach

**DOI:** 10.1186/1940-0640-8-5

**Published:** 2013-03-05

**Authors:** Amanda M Barbeau, Jennifer Burda, Michael Siegel

**Affiliations:** 1 Department of Community Health Sciences, Boston University School of Public Health, 801 Massachusetts Avenue, 3rd Floor, Boston, MA 02118, USA

**Keywords:** Smoking, E-cigarettes, Addiction, Smoking cessation, Qualitative research, Focus group

## Abstract

**Background:**

Nicotine is widely recognized as an addictive psychoactive drug. Since most smokers are bio-behaviorally addicted, quitting can be very difficult and is often accompanied by withdrawal symptoms. Research indicates that nicotine replacement therapy (NRT) can double quit rates. However, the success rate for quitting remains low. E-cigarettes (electronic cigarettes) are battery-powered nicotine delivery devices used to inhale doses of vaporized nicotine from a handheld device similar in shape to a cigarette without the harmful chemicals present in tobacco products. Anecdotal evidence strongly suggests that e-cigarettes may be effective in helping smokers quit and preventing relapse, but there have been few published qualitative studies, especially among successful e-cigarette users, to support this evidence.

**Methods:**

Qualitative design using focus groups (N = 11); 9 men and 2 women. Focus groups were conducted by posing open-ended questions relating to the use of e-cigarettes, comparison of effectiveness between NRTs and e-cigarettes, barriers to quitting, and reasons for choosing e-cigarettes over other methods.

**Results:**

Five themes emerged that describe users’ perceptions of why e-cigarettes are efficacious in quitting smoking: 1) bio-behavioral feedback, 2) social benefits, 3) hobby elements, 4) personal identity, and 5) distinction between smoking cessation and nicotine cessation. Additionally, subjects reported their experiences with NRTs compared with e-cigarettes, citing negative side effects of NRTs and their ineffectiveness at preventing relapse.

**Conclusion:**

These findings suggest tobacco control practitioners must pay increased attention to the importance of the behavioral and social components of smoking addiction. By addressing these components in addition to nicotine dependence, e-cigarettes appear to help some tobacco smokers transition to a less harmful replacement tool, thereby maintaining cigarette abstinence.

## Background

Nicotine is widely recognized as an addictive psychoactive drug [1]. Since most smokers are bio-behaviorally addicted, quitting can be very difficult and is often accompanied by withdrawal symptoms such as anxiety and irritability. Additionally, many smoking cessation products focus on the neuropharmacology of nicotine but fail to address the bio-behavioral component that is heavily ingrained in most addictive practices [2]. As a result, smoking cessation may be unsuccessful, even when there is a strong desire to quit.

Research indicates that smoking cessation medications and nicotine replacement therapy (NRT) can double quit rates [3]. However, even with the use of medications, the success rate for quitting remains low. The percentage of smokers who relapse within six months with the use of NRT is reported to be 93% [4]. Although many NRT products have been available in the US during the past decade, the overall quit rate has changed very little, from 48.7% in 1998 to 51.1% in 2008 [5].

E-cigarettes (electronic cigarettes) are battery-powered nicotine delivery devices. Users inhale doses of vaporized nicotine from a handheld device similar in shape to a cigarette. They are also available in additional shapes, sizes, and vaporized flavors. E-cigarettes deliver nicotine without any combustion or smoke [6,7]. The recent introduction of e-cigarettes to smokers in the US presents a possible new means to enable effective smoking cessation as it addresses the biochemical *and* behavioral aspects of smoking addiction.

One clinical trial of e-cigarettes conducted among smokers with no desire to quit reported a six-month point prevalence smoking cessation rate of 22.5% [8]. An additional 32.5% of smokers reduced their cigarette consumption by at least 50% [8]. In a previously reported survey of a sample of e-cigarette users, it was found that the six-month point prevalence of smoking abstinence was 31%, and respondents who used e-cigarettes over 20 times per day had a quit rate of 70.0%—significantly higher than other smoking cessation methods [9]. Several studies have suggested that merely the presence of smoking behavioral stimuli, even in the absence of nicotine, can reduce the cravings to smoke [10,11]. A number of studies have demonstrated the efficacy of e-cigarettes in alleviating cravings for cigarettes [12,13].

Evidence strongly suggests that e-cigarettes may be effective in helping smokers quit and preventing relapse, but there have been few published studies to explain why this might be the case. A recent study on e-cigarette use was conducted by interviewing individual users; key themes associated with e-cigarette use were identified, such as the culture of “vaping” and the social and informational support among the community [14].

This paper reports a qualitative investigation of the effectiveness of e-cigarettes through focus group discussions among current e-cigarette users. We asked subjects to discuss their perceptions of the efficacy of e-cigarettes for smoking cessation compared to NRTs. This study adds substantially to the current literature on e-cigarettes by helping to identify hypotheses to explain the popularity of these devices and to shed light on the factors which influence the efficacy of different smoking cessation products.

## Methods

### Study design

A qualitative study was conducted using focus group methodology. The study was designed to generate hypotheses regarding the factors that influence the efficacy of smoking cessation aids and to assess the sociocultural and behavioral facets of addiction that the e-cigarette may provide.

### Recruitment

Focus groups were held with a convenience sample of 14 participants recruited by posting an ad on the e-cigarette forums http://www.e-cigarette-forum.com/forum/ and http://www.vapersclub.com. Interested participants responded to the ad via an email address that was only accessible to study recruiters. E-cigarette users, also known as “vapers,” use these websites to discuss topics ranging from e-cigarette brand recommendations to laws about e-cigarettes. E-cigaretteforum.com has 100,000 posts a month and describes itself as the world’s largest e-cigarette forum. Vapersclub.com is the website for the National Vapers Club, a consumer-based organization run and sponsored by vapers that encourages self-regulation by e-cigarette retailers until the federal government develops regulatory standards. The study’s advertisement on the websites contained an explanation of the study’s purpose, estimated time needed to participate, incentives for participation, and a contact email address. Interested individuals replied to the advertisement via a confidential email address. Potential subjects were contacted to determine eligibility for inclusion. Enrollment criteria for the focus groups included being between the ages of 18 and 64, English speaking, past smoker, current e-cigarette user, and able to travel to Boston, MA, to participate. Recruitment took place until two focus groups with 5–7 participants each had registered. This study was reviewed and approved by the Boston University Medical Campus Institutional Review Board, assigned reference number H-29473.

### Participants

The study participants (N = 11) consisted of 9 men and 2 women. There were four participants aged 18–24 years, four participants aged 25–44 years, and three participants aged 45–64 years. Nine of the participants identified themselves as non-Hispanic white. Six participants had some college or an associate degree, three had a four-year college degree, and two had a graduate degree. Their smoking histories varied. Three participants smoked for one to five years, one smoked for five to 10 years, and six smoked from 10 to 40 years. Ten out of the 11 participants were not current users of regular cigarettes. All but two participants smoked at least a half pack of cigarettes per day before using e-cigarettes. Smoking history and exposure to e-cigarettes and other NRTs was determined by completing a survey. The survey asked a series of questions about current smoking status, smoking history, and e-cigarette and tobacco cigarette usage, in addition to asking about smoking status as of February 2010. No participants were excluded from the study due to an inability to drive to Boston, because it was clearly stated in the ad that the focus group was to take place in Boston. Thus we can reasonably assume those who could not travel to Boston to attend the focus group did not inquire about the study.

### Data collection

Subjects read and signed a study consent form before participating in a focus group session. Participants also filled out a short anonymous survey outlining their smoking history and history of e-cigarette use. Two investigators were present at both focus groups. One investigator moderated open-ended questions relating to the use of e-cigarettes, how effective they are with quitting smoking compared with other approaches, barriers to quitting, and reasons for choosing e-cigarettes over other methods. The second investigator took notes. The focus groups were recorded with a digital voice recorder for quality assurance, and participants were assigned and referred to by numbers in order to remove personal identifiers and protect their identity. The focus group notes did not contain personal identifiers. Both focus groups lasted approximately 90 minutes.

### Data analysis

The purpose of our study was to gain qualitative insight into whether e-cigarettes were effective aids in long-term smoking cessation and why this might be the case, in addition to gaining a greater understanding of their efficacy on reducing cravings and preventing smoking relapse. Therefore, we used grounded theory as the conceptual framework to develop theories and explanations for the data [15]. Grounded theory focuses on generating theory instead of using a particular theoretical content that focuses on an element of human experience, like culture or interpretations, to explain the data [16]. Similar to McQueen et al.’s research with vapers [14], we did not develop theories or hypotheses to explain why e-cigarettes may be effective for quitting smoking prior to conducting the research. We developed broad research questions prior to the focus groups but allowed the data to develop themes and explanations as grounded theory suggests.

After the two focus group recordings were transcribed, the transcripts were coded for major themes. The two study investigators read through both transcripts separately and assigned each sentence or paragraph a descriptive and interpretive code rather than using computer software to identify themes. Investigators met to compare codes and discussed themes in order to generate theory topics to further analyze, wrote down key points that were raised in the focus groups, and then identified central themes. The coded transcripts and key point research documents were compared to ensure that all points discussed in the focus groups were acknowledged in the themes and theory developed from the data, and that all investigators agreed with the thematic outcomes. The two groups themes were concordant, and the five themes generated were observed in both groups, indicating that saturation of themes was achieved.

The rationale for conducting a small focus group study was a limitation in study funding and access to e-cigarette users. This study was meant to serve as a small sampling of e-cigarette users, and the data obtained to be used to develop possible hypotheses for testing the effectiveness of e-cigarettes in larger studies. We used two focus groups instead of one because we wanted at least 10 participants and we wanted to be able to compare themes generated by two distinct groups.

## Results

Five main themes were identified that explain why e-cigarettes appear, at least anecdotally, to be efficacious in helping tobacco users quit smoking. Table 1 provides an outline of the themes identified and some examples of narratives expressed by focus group participants illustrating those themes. Additionally, focus group members discussed the perceived efficacy of e-cigarettes compared with conventional NRTs (e.g., nicotine patch, nicotine gum).

**Table 1:** Identified themes and examples of narratives expressed by focus group participants

**Themes**	**Bio-behavioral feedback**	**Social benefits**	**Hobby elements**	**Personal identity**	**Difference between smoking cessation and nicotine cessation**
**Narratives**	“That feeling when it comes down and hits your throat and you inhale it, that’s like a big deal for us all.”	“Going to the website you start hearing people’s stories […] you research until you find something and I kept coming back to this and really liked it. There’s a big support community ethic, which is part of it.”	“I learned about […] the different bases and juices. There’s so much knowledge out there and I became a nerd. And it became a hobby.”	“You know, for years, I loved being able to carry around my pack of cigarettes and my Red Sox lighter. I miss carrying my Red Sox lighter […] it becomes who you are. It becomes, you don’t do anything without a cigarette in your hand. Now I can still do that and still get the nicotine without disgusting somebody else because I am smoking, and it does stink.”	“When I first started, that was the plan. But I enjoy it now. I don’t see anything wrong with it.”
					“My goal is to be nicotine free at some point but I’m not in a hurry, either.”
	“[…] When I quit cigarettes, my fixation with, you know, vaping, is very similar. So I like to vape while I’m in the car, I like to vape while after I have a meal or when I have a coffee or when I’m drinking and so on, so it mirrors that almost.”				
			“I like all the flavors, I like the devices. You know, it’s my new hobby, my new collection. I don’t collect lighters now, I’m collecting juice and devices.”		
		“Having the support was instrumental.”			
		“You don't hear about two people on the patch talking about their patches or what brand their trying or what not.”			
			“Perfect vape.”		
				Refer to themselves as “vapers.”	

### Theme: bio-behavioral feedback

Participants in both focus groups felt that e-cigarette vaping mimicked smoking a real cigarette. The e-cigarette addressed participants’ oral fixation as well as the experience of inhaling, feeling the smoke hit the back of the throat (“throat hit”), and seeing the vapor cloud when exhaling. Participants emphasized the significance of the throat hit and vapor cloud, “That feeling when it comes down and hits your throat and you inhale it, that’s, like, a big deal for us all.” Additionally, the e-cigarette users followed their regular cigarette routine when vaping: “[…] When I quit cigarettes, my fixation with, you know, vaping, is very similar. So I like to vape while I’m in the car, I like to vape after I have a meal, or when I have a coffee, or when I’m drinking, and so on; so it mirrors that, almost.” Participants explained that they were able to swap e-cigarettes into their normal everyday smoking routine.

### Theme: social benefits

The notion of a vaping community was continually reiterated among participants. They pointed out the significance of having the online community forums where they could ask questions and find support and encouragement from fellow users. One participant shared, “Going to the website, you start hearing people’s stories […] you research until you find something, and I kept coming back to this and really liked it. There’s a big support community ethic, which is part of it.” Another participant shared, “Having the support [from other e-cigarette users] was instrumental.” In addition to the large support network in the vaping community, enjoying the social aspect of e-cigarettes was noted. There are vaping clubs where e-cigarette users can vape together and hold discussions. As one participant mentioned, “You don’t hear about two people on the patch talking about their patches or what brand their trying or what not.” Participants enjoyed the sense of community and support from other vapers.

### Theme: hobby elements

The participants repeatedly discussed vaping as a hobby. Most of them didn’t necessarily see e-cigarettes as a means to quit nicotine altogether but liked the experience in addition to mixing and matching different types of e-cigarette parts and “juice” flavors. One participant described it as a hobby: “I learned about […] the different bases and juices. There’s so much knowledge out there, and I became a nerd. And it became a hobby.” Another person shared, “I like all the flavors, I like the devices. You know, it’s my new hobby, my new collection. I don’t collect lighters now, I’m collecting juice and devices.” Participants enjoyed the autonomy of playing with the different components of e-cigarette to find their “sweet spot” or “perfect vape.”

### Theme: personal identity

The majority of participants identified themselves as “vapers.” Previously they had defined themselves as “smokers,” and the e-cigarette allowed them to redefine their identity. According to one former smoker, “You know, for years, I loved being able to carry around my pack of cigarettes and my Red Sox lighter. I miss carrying my Red Sox lighter […] it becomes who you are. It becomes, you don’t do anything without a cigarette in your hand. Now, I can still do that and still get the nicotine without disgusting somebody else because I am smoking, and it does stink.” Instead of identifying as smokers and their brand of cigarettes, they now discuss and identify themselves by the type of e-cigarette they use and flavors they like.

### Theme: difference between smoking cessation and nicotine cessation

As mentioned in the hobby elements theme, many of the participants did not necessarily see e-cigarettes as a means to transition to quitting nicotine altogether. Participants emphasized the difference between smoking cessation and nicotine cessation. E-cigarettes allowed them to quit smoking, but some participants did not want to quit nicotine, because they enjoy the e-cigarette experience and viewed it as going from a dangerous form of nicotine intake in cigarettes to a safer form in e-cigarettes. When asked about lowering the nicotine levels to the possibility of zero, one participant responded, “When I first started, that was the plan. But I enjoy it now. I don’t see anything wrong with it.” For those intending to eventually quit using e-cigarettes, the sense of urgency of needing to quit is not the same as with regular cigarettes. One participant shared, “My goal is to be nicotine-free at some point, but I’m not in a hurry, either.” Participants also discussed how the NRTs they were familiar with (patch, gum, etc.) were meant to be temporary and to eventually wean people off of nicotine all together, whereas reducing nicotine dependence is optional with e-cigarettes.

### Perceived efficacy of e-cigarettes versus conventional nicotine replacement therapies

In the first focus group, three of six participants had tried varenicline, four of six had tried nicotine gum, and five of six had tried the nicotine patch. In the second focus group, participants expressed little confidence in the perceived efficacy of conventional NRTs, claiming that they still found themselves craving cigarettes while using these methods. They also reported undesirable side-effects and many quit attempts using NRTs that resulted in relapse to cigarette use. One participant reported that, while on the patch, “If you’re asking what do we mean when we say the patches didn’t work, at a certain point in time I was having a cigarette after; so whether it was a failure of the patch, or psychological, or with me or us, it does seem at the end of the day or end of the month you’re back on the cigarettes.” Another participant reported that the patch satiated the physiological craving for nicotine but not the psychological: “[…] The patch was able to satisfy the physical craving for me. It’s the psychological craving.”

Negative side-effects were reported during the use of NRTs. Some claimed to experience negative side-effects with the gum and the patch, with one participant suffering from hiccups during the use of the gum: “You get the hiccups. You feel your heart going like crazy.” Others reported extremely disturbing dreams while taking varenicline. One participant even claimed to smoke cigarettes while using varenicline: “I smoked through the whole thing. I was just smoking and taking varenicline.” When asked how e-cigarettes compare with traditional NRTs, one participant stated, “It’s the only thing that ever worked. I think it’s part the act. I think its part the way it’s delivered.” And another, speaking about NRTs, stated, “The delivery doesn’t work, that’s what I learned. Plain and simple.”

## Discussion

Results from these focus groups add to the albeit still limited research base regarding e-cigarettes and their usefulness as smoking cessation tools. The information gained provides new insights into the social and group dynamics that may underlie the reasons why NRT has such low observed rates of effectiveness, and why e-cigarettes, at least anecdotally, appear to be more effective for many vapers. Most notably, these include e-cigarettes becoming part of the vaper’s social identity, the recognition of vaping as a hobby, and the ability of these devices to aid in smoking cessation without complete nicotine cessation.

These insights suggest that health practitioners should pay increased attention to the behavioral and social components of smoking addiction, many of which are not addressed by conventional NRTs, as noted by participants in this study. Greater understanding of these components could lead to more effective approaches to treating cigarette addiction, as the e-cigarette users in our focus groups experienced alleviation of withdrawal symptoms and achieved smoking cessation more effectively than they had with conventional NRT.

The ability for e-cigarette users to redefine themselves from “smokers” to “vapers” could be incredibly useful not only in helping tobacco smokers transition to a less harmful replacement tool but also in helping them maintain cigarette abstinence. Many participants in the focus groups reported having relapsed multiple times using the patch, nicotine gum, and prescription medications. This sense of identity as a vaper, both on an individual and group level, appears to give e-cigarette users a sense of ownership over their cigarette addiction. This identity also appeared to be formed and reinforced through the support provided by e-cigarette online forums, where e-cigarette users exchanged information, displayed pride over number of days cigarette-free, and received encouragement for quitting [14].

E-cigarette use being described as a hobby suggests that the experience is enjoyable and that having a variety of flavors, devices, and nicotine levels available reinforces the motivation to quit smoking and helps prevent relapse. However, due to this variety, further investigation into the concept of the learning curve that occurs with e-cigarette use is warranted.

Both groups emphasized the difference between smoking cessation and nicotine cessation and viewed the e-cigarette as being a safer form of nicotine delivery. Participants recognized that their addiction to nicotine had not subsided, but the means for nicotine administration was replaced by a perceived safer alternative.

The perceptions of e-cigarette users towards vaping as compared to smoking are relevant to legal and policy considerations regarding these products. The subjects in our study clearly viewed e-cigarettes as both a tool for smoking cessation and a safer alternative to cigarettes. However, the current legal and policy framework surrounding e-cigarettes precludes their being marketed with claims that they are safer than regular cigarettes or that they may be useful in smoking cessation. The former claim might be considered a reduced-risk claim under the Family Smoking Prevention and Tobacco Control Act [17], and the latter might be considered a therapeutic claim, which would put the product under the scrutiny of the US Food, Drug, and Cosmetic Act [18]. Ironically, although e-cigarettes contain no tobacco, the courts have ruled that these products must be regulated as tobacco products rather than as drugs [19]. The way in which actual users of e-cigarettes perceive these products should be considered by the US Food and Drug Administration, which is currently developing regulations for e-cigarettes.

This study was limited in that the sample of participants was not representative of all e-cigarette users; they were recruited from only two online forums and included only participants who were willing and able to drive to the focus group location. Furthermore, the sample represents e-cigarette users who were committed and involved enough with e-cigarettes to be on these forums. This presents an inherent bias in the sample, as those who participated in the focus groups likely favored e-cigarettes for smoking cessation. Therefore, the information gained within the focus groups may not be generalizable to e-cigarette users overall, and this inherent bias could lead to an overestimation of the successful use of e-cigarettes as smoking cessation tools. Although it is true that sampling bias exists, we do not believe this threatens the validity of our conclusions, as this study was intended to bring to light how e-cigarettes are perceived among those who have found them helpful. Further research with larger sample sizes from multiple sites would yield a greater representation of the e-cigarette user population, as would the inclusion of previous e-cigarette users who relapsed.

## Conclusion

There is anecdotal evidence that e-cigarettes may be useful in helping smokers quit, but little is known about the reasons why these products help smokers achieve cessation or how smokers perceive these products in comparison to other cessation strategies such as traditional NRTs. We conducted focus groups with e-cigarette users to assess their perceptions of the efficacy of these devices in smoking cessation compared with other strategies such as varenicline, nicotine gum, and the nicotine patch. We identified five major themes to explain why e-cigarettes appear to be helpful in aiding cessation, at least for some users. These themes highlight the need for health practitioners and policy makers to give greater consideration to the physical, behavioral, and social aspects of cigarette smoking addiction and not merely to treat smoking addiction as a pharmacologic addiction to nicotine.

## Competing interests

The authors declare that they have no competing interests.

## Authors’ contributions

All authors participated in the conception of the study and in crafting the research design. AB and JB prepared the IRB protocol, conducted the focus group sessions, and analyzed the focus group transcripts. All authors participated in the review and interpretation of the data, the preparation of the manuscript, and the review of the manuscript for critical content. All authors read and approved the final manuscript.

## References

[B1] US Centers for Disease Control How tobacco smoke causes disease—the biology and behavioral basis for smoking-attributable disease fact sheethttp://www.surgeongeneral.gov/library/tobaccosmoke/factsheet.html 21452462

[B2] Buchhalter AR Acosta MC Evans SE Breland AB Eissenberg T Tobacco abstinence symptom suppression: the role played by the smoking-related stimuli that are delivered by denicotinized cigarettes Addiction 2005 100 550 559 10.1111/j.1360-0443.2005.01030.x 15784070

[B3] US Department of Health & Human Services Treating tobacco use and dependence: clinical practice guideline http://www.cdc.gov/mmwr/PDF/wk/mm5844.pdf

[B4] Hughes JR Shiffman S Callas P Zhang J A meta-analysis of the efficacy of over-the-counter nicotine replacement Tob Control 2003 12 21 27 10.1136/tc.12.1.21 12612357 PMC1759113

[B5] US Centers for Disease Control Cigarette smoking among adults and trends in smoking cessation—United Stateshttp://www.cdc.gov/mmwr/preview/mmwrhtml/mm5844a2.htm

[B6] US Food and Drug Administration E-cigarettes: questions and answers www.fda.gov/ForConsumers/ConsumerUpdates/ucm225210.htm

[B7] Stead L Perera R Bullen C Mant D Lancaster T Nicotine replacement therapy for smoking cessation Cochrane Database Syst Rev 2012 11 CD000146 18253970 10.1002/14651858.CD000146.pub3

[B8] Polosa R Caponnetto P Morjaria JB Papale G Campagna D Russo C Effect of an electronic nicotine delivery device (e-cigarette) on smoking reduction and cessation: a prospective 6-month pilot study BMC Public Health 2011 11 786 10.1186/1471-2458-11-786 21989407 PMC3203079

[B9] Siegel M Tanwar K Wood K E-cigarettes as a smoking cessation tool Am J Prevent Med 2011 40 4 472 475 10.1016/j.amepre.2010.12.006 21406283

[B10] Dar R Rosen-Korakin N Shapira O Gottlieb Y Frenk H The craving to smoke in flight attendants: relations with smoking deprivation, anticipation of smoking, and actual smoking J Abnorm Psychol 2010 119 1 248 253 20141262 10.1037/a0017778

[B11] Barrett SP The effects of nicotine, denicotinized tobacco, and nicotine-containing tobacco on cigarette craving, withdrawal, and self-administration in male and female smokers Behav Pharmacol 2010 21 2 144 152 10.1097/FBP.0b013e328337be68 20168213

[B12] Bullen C McRobbie H Thornley S Glover M Lin R Laugesen M Effect of an electronic delivery device (e-cigarette) on desire to smoke and withdrawal, user preferences and nicotine delivery: randomized cross-over trial Tob Control 2010 19 98 103 10.1136/tc.2009.031567 20378585

[B13] Cahn Z Siegel M E-cigarettes as harm reduction strategy for tobacco control: a step forward or a repeat of past mistakes? J Public Health Policy 2011 32 16 31 10.1057/jphp.2010.41 21150942

[B14] McQueen A Tower S Sumner W Interviews with “vapers”: implications for future research with e-cigarettes Nicotine Tob Res 2011 13 860 867 10.1093/ntr/ntr088 21571692

[B15] Bradley EH Curry LA Devers KJ Qualitative data analysis for health services research: developing taxonomy, themes, and theory Health Serv Res 2007 42 1758 1772 10.1111/j.1475-6773.2006.00684.x 17286625 PMC1955280

[B16] Patton MQ Qualitative Research and Evaluation Methods 2011 3 Thousand Oaks, CA: Sage Publications

[B17] Family Smoking Prevention and Tobacco Control Acthttp://www.fda.gov/tobaccoproducts/guidancecomplianceregulatoryinformation/ucm246129.htm

[B18] 21 US Code Chapter 9: Federal Food, Drug, and Cosmetic Act http://uscode.house.gov/download/pls/21C9.txt

[B19] United States Court of Appeals for the District of Columbia Circuit Sottera, Inc. v. US Food and Drug Administration http://www.cadc.uscourts.gov/internet/opinions.nsf/D02F9D2CA50299F0852577F20070BCC2/$file/10-5032-1281606.pdf

